# Focal muscle vibration and neurocognitive exercise improve function and reduce neuropathic pain after sciatic nerve injury: a case report assessment through gait analysis

**DOI:** 10.3389/fresc.2025.1628749

**Published:** 2025-07-22

**Authors:** Filippo Camerota, Naomi Francesca Pocino, Federico Zangrando, Alessia Lucani, Marco Paoloni, Massimiliano Mangone, Claudia Celletti

**Affiliations:** ^1^ Physical Medicine and Rehabilitation Division, Umberto I University Hospital, Rome, Italy; ^2^ Department of Anatomical and Histological Sciences, Legal Medicine and Orthopedics, Sapienza University, Rome, Italy; ^3^ Department of Life Sciences, Health, and Health Professions, Link Campus University, Rome, Italy

**Keywords:** focal muscle vibration, gait analysis, neuropathic pain rehabilitation, neurorehabilitation, peripheral nerve lesion, sciatic nerve

## Abstract

The sciatic nerve may be injured during total hip arthroplasty (THA). This complication can lead to severe neuropathic pain syndrome. This case report aims to investigate the effect of a combined treatment approach involving neurocognitive rehabilitation and focal mechanical vibrations in a patient affected by iatrogenic sciatic nerve injury and neuropathic pain following total hip arthroplasty (THA). The patient was followed over a total of 1 year, during which she underwent three cycles of 12-week neurocognitive physiotherapy, with weekly 1 h sessions, interspersed with two cycles of 1-week therapy involving only focal mechanical vibrations (fMV). She was also evaluated with a clinical scale and gait analysis. We have observed a significant reduction in the pain perceived by the patient, although not complete, but interestingly, the patient reported resolution of allodynia right after the first fMV session. Furthermore, the duration of the gait cycle approached more normal values. Overall, the combined treatment of neurocognitive rehabilitation and focal mechanical vibrations yielded positive results and contributed significantly to the reduction of chronic neuropathic pain in the patient. Simultaneously, the focal mechanical vibrations seem to provide crucial proprioceptive stimulation, promoting better motor control and further aiding in neuropathic pain reduction.

## Introduction

Mononeuropathy is a peripheral neuropathy that involves a single nerve; among them, the sciatic nerve may be injured by direct trauma, pelvic fractures, and injections administered in the gluteal region. Additionally, it may be of interest as a rare complication of total hip arthroplasty (THA) with incidences of 0.08%–7.6%. Risk factors include developmental hip dysplasia, female sex, and revision surgery (1, 2).

No universally accepted therapeutic algorithm has been established in the literature. However, documented management approaches of sciatic nerve injuries include surgical exploration, in cases of neuropathic pain or confirmed hematoma, and rehabilitative treatment (2).

Focal vibration is an increasingly utilized therapeutic approach in the rehabilitation of both orthopedic and neurological conditions. Its application is underpinned by its ability to reduce spasticity, facilitate motor control, and activate proprioceptors, thereby promoting neuromuscular optimization (3). Emerging studies support the hypothesis that focal mechanical vibration (fMV) may be a valuable technique to incorporate within a multimodal approach for the treatment of pain symptoms. Initially, this assumption was primarily based on the gate control theory; however, current perspectives increasingly refer to neuromodulation involving mechanisms of neural signal suppression or facilitation (4).

This study aims to investigate the effects of a combined approach of neurocognitive rehabilitation and focal mechanical vibration therapy on neuropathic pain secondary to sciatic nerve injury. Additionally, it seeks to assess whether this treatment can provide beneficial effects in terms of functional recovery, gait abilities, and patient quality of life.

## Case report

In this case report, we describe the clinical case of a 69-year-old female patient with a history of chronic neuropathic pain due to a prior diagnosis of right sciatic nerve mononeuropathy, confirmed through electromyographic and electroneurographic studies, secondary to right total hip arthroplasty (THA). The patient underwent THA via a posterolateral approach in June 2022 following a diagnosis of severe osteoarthritis of the right hip. Her medical history was significant for hypertension and osteoporosis, both under pharmacological treatment.

At the time of discharge from the surgical unit, 10 days postoperatively, the patient presented with a deficit in ankle and toe dorsiflexion on the right side. She subsequently underwent an inpatient rehabilitation program followed by multiple cycles of physiotherapy until September 2023, with no significant improvement in dorsiflexion recovery. Consequently, an orthotic device for foot drop was prescribed.

In the same month, 14 months after surgery, the patient presented to our outpatient clinic due to persistent motor impairment and long-standing neuropathic pain, which had remained unchanged for several months. Gabapentin had been previously prescribed for pain management, but the patient refused to take it due to reported adverse effects.

On clinical examination, the patient exhibited preserved right plantar flexion, minimal activation of the peroneal muscles during eversion (Medical Research Council Grade 2), and complete absence of contraction of the tibialis anterior and toe extensor muscles. She exhibited allodynia throughout the right lower limb and anesthesia of the ipsilateral toes. Hypesthesia was noted in the L4–L5 dermatome.

Ambulation examination revealed a steppage gait pattern, prolonged stance phase on the left foot, and reduced step length and walking speed. The patient was able to ambulate only with the assistance of a left-sided Canadian crutch and an orthotic device for the right ankle.

At the time of the initial visit, the patient had already undergone three separate electroneurographic and electromyographic (ENG/EMG) assessments. The first examination, performed 1 month after surgery, documented denervation activity (positive sharp waves and fibrillations) in the right tibialis anterior and medial gastrocnemius, consistent with an acute phase axonotmesis of the right sciatic nerve. The second EMG/ENG follow-up (FU), conducted 5 months later, continued to reveal severe impairment of the lateral component of the right sciatic nerve, with a slight increase in compound muscle action potential amplitude. Approximately 10 months after the iatrogenic injury, the third evaluation demonstrated the emergence of increased insertional activity in the right tibialis anterior muscle.

## Methods

**Figure 1:**
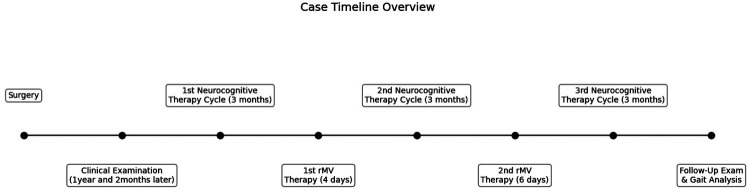
Case report timeline overview.

The patient was evaluated with clinical scale and gait analysis: before treatment (T0), after the first period of vibratory therapy (T1), after the second fMV treatment (T2), and at follow-up (FU) conducted 3 months after the completion of all treatments. During the entire period, she underwent a specific rehabilitation treatment. A case report timeline is shown at Figure 1.

### Vibratory therapy

FMV was provided using a specific device consisting of an electromechanical transducer, a mechanical support, and an electronic control device (CRO SYSTEM; NEMOCO srl, Italy).

The mechanical support enabled the orientation, positioning, and rigid fixation of the transducer in every direction relative to the patient's body. The transducer was positioned perpendicularly over the belly of the target muscle.

Two vibratory therapy cycles were administered: a four daily session and six daily session 3 months apart. Target muscles included the gluteus medius, quadriceps, biceps femoris, anterior tibialis, and soleus, with the plantar fascia also treated for localized pain. Each muscle was treated for 20 min, except for the quadriceps, which was treated for 10 min. Therapy involved 100 Hz mechanical vibrations targeting proprioceptors to stimulate Ia afferents without triggering tonic vibration reflexes. When possible, a minimal intermittent contraction was requested to optimize the response to vibration therapy.

### Rehabilitation treatment

The rehabilitation treatment was carried out over a total period of 10 months. A 1-week interval was scheduled every 3 months, during which the patient underwent focal mechanical vibratory therapy sessions. Physiotherapy sessions were conducted weekly, for a total of 12 sessions per cycle, each lasting 60 min. To reduce pain, the exercises initially involved sensory stimulation through tactile, kinesthetic, pressure, and weight discrimination tasks; therefore, the use of motor imagery was introduced to encourage voluntary contractions. In this way, the neurocognitive approach contributed to inducing neuronal plasticity and motor relearning (5). Among the proposed exercises, there were those aimed at tactile recognition of various areas touched by the therapist, identification of different foot positions, and differentiation of various textures placed under the sole of the foot. Subsequently, the patient progressed to weight-shifting exercises in a standing position using balance scales, as well as gait training on surfaces of varying consistency.

### Gait analysis

To properly assess gait function, an instrumented gait analysis evaluation was performed at T0, T1, T2, and FU comprehensive of kinematic, kinetic, and surface EMG assessment.

Gait analysis was conducted using a 3D optoelectronic system (Smart D500; BTS Bioengineering), a force platform (Kistler), and two TV camera video systems (BTS Bioengineering). Markers were positioned on the participant's body, as described by Davis et al. (6). Spatiotemporal parameters, including phase durations, step length, cadence, and speed, were recorded.

## Results

Throughout the entire duration of our rehabilitation management, the patient did not experience any adverse effects related to the treatment. A gradual improvement in pain symptoms was observed, with resolution of allodynia immediately following the first session of focal muscle vibration therapy. Below, we provide a detailed analysis of the results obtained across the different domains.

### Pain

On the NRS scale, pain decreased from 6 at T0 to 3 by T1, remaining stable thereafter. The McGill pain questionnaire (MPQ) scores showed a gradual decrease in total pain indices, except for the pain rating index sensory (“PRI S”) score which initially increased and then decreased from T2 onwards, reflecting initial somatosensory reconditioning and improved body awareness through therapy. Detailed results at MPQ are shown in Table 1*.*

**Table 1:** McGill pain questionnaire and Short Form-36 results.

MPQ	T0	T1	T2	FU
PRI S	29.1	30.3	21.1	22.9
PRI A	10.7	10.1	10.1	10.3
PRI E	2.1	2.1	2.1	2.1
PRI M	13.3	10.3	11.7	7.8
P tot	55.2	52.8	45	43.1
P S/A	2.53	4.73	2.09	2.91
NWC	18	18	16	16
Physical functioning	40	–	–	60
Role limitations due to physical health	0	–	–	100
Role limitations due to emotional problems	66.7	–	–	0
Vitality	20	–	–	45
Mental health	52	–	–	68
Social functioning	50	–	–	50
Pain	45	–	–	45

PRI S, pain rating index sensory; PRI A, pain rating index affective; PRI E, pain rating index evaluative; PRI M, pain rating index miscellaneous; TOT, total; P S/A, sensory/affective; NWC, number of words chosen.

Pain assessments revealed substantial improvement throughout the treatment, with a marked decrease in perceived pain, although not entirely resolved. Initial vibratory therapy (fMV) session eliminated allodynia in the leg and alleviated foot pain, substantiating the effectiveness of focal mechanical vibrations. The neuromuscular spindle and mechanoreceptor stimulation likely facilitated pain reduction through mechanisms such as non-nociceptive fiber activation, somatosensory reorganization via cortical plasticity, and modulation of descending inhibitory pathways enhancing inhibitory neurotransmitter release (7).

### Gait analysis and EMG/ENG

After treatment, gait cycle asymmetry persisted, though it was reduced. The stance phase discrepancy between limbs improved, while cadence increased from 70.8 steps/min at T0 to 86.1 steps/min at FU. Step length and walking speed also improved. Table 2 shows gait analysis parameters during treatment.

**Table 2:** Gait analysis at T0, T1, T2, and FU.

Gait analysis	T0		T1		FU		Standard
	dx	sx	dx	sx	dx	sx	
Cycle duration (s)	1.69	1.73	1.56	1.66	1.46	1.34	1.1
Stance duration (s)	1.02	1.19	0.86	1.08	0.94	0.93	0.65
Swing duration (s)	0.66	0.54	0.7	0.57	0.52	0.41	0.44
Stance phase (%)	61.21	68.7	55.08	65.42	64.38	69.82	58.98
Swing phase (%)	38.78	31.21	44.92	34.58	35.62	30.18	40.03
Single support (%)	32.02	37.96	36.64	42.46	28.02	39.04	38.87
Double support (%)	14.14	13.19	15.61	8.54	18.98	10.51	10.27
Average speed (m/s)	0.3		0.3		0.4		1.2
Average speed (%)	17.25		16.02		25.9		80
Cadence (steps/min)	70.8		75		86.1		114
Cycle length (m)	0.5	0.45	0.41	0.42	0.59	0.59	1.36
Cycle length (%)	30.77	27.8	25.41	25.95	35.9	36.3	80
Step length (m)	0.28	0.19	0.26	0.16	0.31	0.28	0.62
Step width (m)	0.22		0.22		0.18		0.08

Gait analysis demonstrated significant enhancements during treatment and at follow-up. In fact, following the initial cycle of vibratory therapy, the patient discontinued use of the Codivilla spring, regaining stability during ankle dorsiflexion. By FU, there was complete recovery of dorsiflexion at heel strike, improved function of the gluteus medius, and symmetrical support phases, culminating in a more natural and harmonious gait. Improvements in stride length, speed, and cadence reduced compensatory patterns, enabling reduced fall risk.

At the end of the treatment, the patient underwent a follow-up EMG/ENG assessment, which revealed improved motor unit recruitment at maximal effort in the right leg musculature. Moreover, motor unit potentials in the right tibialis anterior muscle exhibited a complex morphology, with a duration >10 ms and an amplitude >2 mV. In contrast, the pretreatment assessment had documented polyphasic potentials with suboptimal duration and amplitude.

### Quality of life

**Figure 2:**
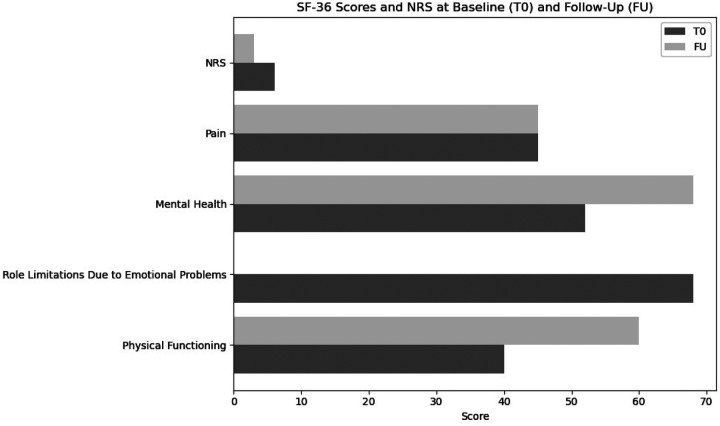
Short Form-36 and NRS scores at T0 (first examination) and FU (follow-up).

Finally, the Short Form-36 (SF-36) outcomes indicated notable gains in physical functioning and role limitations due to physical health (from 40–60 to 0–100, respectively), although emotional well-being and the affective PRI domains showed only minimal progress. We suggest that this finding underscores the importance of integrating psychological support within rehabilitation teams to address emotional health comprehensively. SF-36 scores are shown in Figure 2.

## Discussion

This case report illustrates the potential benefits of combining neurocognitive rehabilitation with focal mechanical vibration (fMV) in managing chronic neuropathic pain and associated gait impairments. The patient, despite undergoing traditional physiotherapy for 2 years without improvements, exhibited significant pain reduction, improved ambulation, and enhanced quality of life following the integrated intervention. These findings support the notion that chronic neuropathic pain, even in postsurgical contexts with long-standing deficits, may remain modifiable through targeted neurorehabilitative strategies.

From a mechanistic perspective, the effectiveness of fMV likely stems from its ability to engage multiple neurophysiological pathways. Unlike traditional physiotherapy, focal vibration directly stimulates mechanoreceptors, particularly primary endings of muscle spindles (Ia afferents), thereby enhancing proprioceptive feedback and modulating central sensory processing (8). This activation leads to reorganization of sensorimotor circuits and promotes cortical plasticity, which plays a pivotal role in functional motor recovery.

Several studies have demonstrated that vibration stimuli can induce long-term potentiation-like effects in the somatosensory cortex, contributing to enhanced sensorimotor integration (7). Moreover, proprioceptive input from vibration may inhibit nociceptive transmission at both spinal and supraspinal levels via mechanisms involving wide dynamic range neurons and descending inhibitory pathways (3). While early models attributed this analgesic effect to the “gate control theory” (9), more recent neuroimaging and neurophysiological data suggest a more complex interaction between the somatosensory and pain matrix, including the insular cortex, anterior cingulate cortex, and prefrontal regions (10).

The therapeutic response suggests that central plasticity remains accessible to modulation beyond the subacute phase. Additionally, we report that the patient did not undergo any changes in pharmacological treatment during care. Furthermore, the perceived improvement in gait allowed her to discontinue the use of the right ankle orthosis previously prescribed.

From the patient's perspective, emotional well-being showed only partial improvement during the treatment, remaining a limiting factor in social interactions and daily functioning. Although some progress was initially reported—particularly in the domain of role limitations due to emotional problems in the SF-36 test—a decline at follow-up suggests a lack of sustained benefit. This highlights the importance of the therapeutic alliance and ongoing support provided by the rehabilitation team, which the patient perceived as valuable during treatment. Specific attention was given to her concerns, including perceived gait instability and associated emotional distress, contributing to improved self-confidence in social contexts.

However, the deterioration observed at follow-up underscores the need for psychological support from specialized professionals to ensure long-term emotional and psychological outcomes, as well as a potentially greater reduction in pain.

However, the study's limitations must be acknowledged. As a single-case report, its generalizability is limited. Nevertheless, the documented improvements in function and pain perception suggest that future randomized studies are warranted to explore the efficacy of similar interventions across broader cohorts. Stratifying patients based on clinical features such as pain chronicity, proprioceptive deficits, and central sensitization indices may help identify those most likely to benefit.

In conclusion, this case highlights the potential of an integrative neurorehabilitative model combining focal mechanical vibration and neurocognitive strategies to address the complex pathophysiology of chronic neuropathic pain. Clarifying the neurophysiological mechanisms underlying these improvements provides a stronger basis for clinical application and supports the development of tailored rehabilitation protocols in chronic pain management.

## Data Availability

The original contributions presented in the study are included in the article/Supplementary Material; further inquiries can be directed to the corresponding author.
